# Brief Report on the 11th Meeting of the European Forum on Antiphospholipid Antibodies

**DOI:** 10.1007/s11926-019-0805-1

**Published:** 2019-02-11

**Authors:** Walid Chayouâ, Dongmei Yin, Philip G. de Groot, Stéphane Zuily, Denis Wahl, Bas de Laat, Hilde Kelchtermans

**Affiliations:** 1 Synapse Research Institute, Maastricht, The Netherlands; 2 Cardiovascular Research Institute Maastricht, Maastricht University Medical Center, Maastricht, The Netherlands; 3 Université de Lorraine, Inserm, DCAC, Vascular Medicine Division and Regional Competence Center for Rare Vascular and Systemic Autoimmune Diseases, Centre Hospitalier Regional Universitaire de Nancy, 54000 Nancy, France

The European Forum on Antiphospholipid Antibodies provides a multidisciplinary network of diverse experts with a common interest in antiphospholipid antibodies (aPL) and antiphospholipid syndrome (APS). Ten successful meetings have been organized since 1997 in different cities (Paris, Venice, Utrecht, London, Barcelona, Ljubljana, Marseille, Padua, Krakow, and Nancy), providing an excellent opportunity for European clinicians and scientists involved in the field of APS to meet and strengthen current and future collaborations.

The 11th Meeting of the European Forum on Antiphospholipid Antibodies was held in the monumental Lambertus Church in Maastricht, The Netherlands, from September 25 to 26, 2018. The Forum brought together approximately 125 participants from 16 different countries, including the USA, Argentina, and Russia (Fig. 1). Participants with various scientific/medical backgrounds presented an update of their current research in the APS field and reached out to the European Forum for collaboration in new/ongoing projects. Lectures were not only innovative, but also followed by interesting discussions with the audience. This report summarizes the different scientific sessions of the meeting. The abstract book, as well as some of the presentations are available for educational purpose through the website https://apsmaastricht.com.

The current general coordinator of the European Forum on Antiphospholipid Antibodies is Dr. Denis Wahl (Nancy, France). Dr. Bas de Laat (Maastricht, The Netherlands) and Dr. Hilde Kelchtermans (Maastricht, The Netherlands) organized the 11th Forum meeting in Maastricht. The next meeting will take place in Belgrade, Serbia, in the spring of 2020.

## Scientific Session I: Serology of APS

To start off the session, K. Devreese (Ghent, Belgium) presented a summary of laboratory criteria and non-criteria aPL tests with a focus on new tests and techniques. All laboratory criteria aPL suffer from a lack of standardization. Although the new automated systems are more rapid, less labor-intensive, and characterized by a better between-run and inter-laboratory variation, differences in results obtained by the available platforms remain a challenge. Hence, classification of APS patients and follow-up is advised to be performed with the same system. Although promising results were obtained with the non-criteria anti-domain I (aDI) and anti-phosphatidylserine/prothrombin (aPS/PT) assays, the clinical significance needs to be further investigated.

An overview of the risk factors and the available scoring models was presented by S. Sciascia (Turin, Italy). Especially the Global APS Score (GAPSS), taking into account criteria aPL positivity, cardiovascular risk factors and extra-criteria tests proved to be useful for risk stratification and prognosis in several prospective and retrospective studies.

M. Efthymiou (London, UK) and S. Sciascia (Turin, Italy) presented the lupus anticoagulant (LAC) and aPL results from the APS ACTION study, respectively, demonstrating the need for standardization of current criteria assays. The APS ACTION study is a 10-year international prospective study in aPL-positive patients. For the LAC results, the variability was reduced when the same reagent, analyzer, and protocol were used, as done by the five core laboratories. However, local/hospital results were not reliable in 80% of the samples. Anti-cardiolipin (aCL) and anti-β2glycoprotein I (aβ2GPI) results showed good categorical agreement between aPL testing at inclusion and re-test results of the five core laboratories. This agreement further increased when considering high titer samples (> 40 units).

W. Chayouâ (Maastricht, The Netherlands) showed in a multicenter study including 1068 patients that isolated IgM is not associated with thrombosis or pregnancy morbidity and that IgM antibodies within the current aPL-panel do not have an added value in the diagnosis of APS. However, IgM positivity proved to have an added value in risk stratification for thrombotic and pregnancy morbidity. Of note, IgM titers did not differ between diseased and control patients.

R. Urbanus (Utrecht, The Netherlands) investigated the LAC paradox and illustrated that LAC and activated protein C (APC) resistance are two sides of the same coin. Interestingly, aβ2GPI antibodies induce LAC in a Factor V (FV)–dependent manner and LAC proved to result from attenuated FV activation by activated FX (FXa). Furthermore, aβ2GPI-β2GPI complexes bind to FV and induce APC resistance, which is a known risk factor for thrombosis.

In the abstract presentations of session I, P. De Kesel (Ghent, Belgium) demonstrated that both DOAC Stop and activated carbon eliminate the effect of DOACs on LAC tests, except for high therapeutic doses. As DOAC Stop was found to induce clotting time changes in non-anticoagulated samples, possibly leading to false positive LAC results, DOAC Stop should exclusively be applied to samples containing DOACs. In a separate study, heparins and heparinoids were found to concentration-dependently affect LAC testing in vitro. This effect could not be abolished by activated carbon. However, false-positive LAC results were only obtained at supratherapeutic levels.

O. Cabrera-Marante (Madrid, Spain) followed 244 asymptomatic carriers of IgA aβ2GPI antibodies for a period of 5 years. The presence of IgA aβ2GPI antibodies proved to be the main independent risk factor to develop APS, with arterial thrombosis as the most frequent APS event.

M. Serrano (Madrid, Spain) investigated the epitopes recognized by aβ2GPI IgA antibodies. IgA antibodies proved to recognize four peptide zones in DIII, DIV, and DV, located in the same lateral zone and exposed in the J-shape of the β2GPI protein. As these zones were previously shown to be pathogenic in an in vivo model, IgA antibodies can be considered pathogenic per se and should be added to the consensus antibodies.

A. Hoxha (Padua, Italy) explored the role of aPS/PT antibodies as a risk factor of thrombosis in 191 aPL carriers. IgG aPS/PT proved to be an independent risk factor for thrombosis and improved risk stratification when added to the criteria panel. IgM aPS/PT was associated with isolated LAC and may be indicative for a low thrombosis risk.

In a cohort of patients with recent ischemic stroke, A. Serrano (Madrid, Spain) investigated the prevalence of non-criteria aPL. Only 6% of the patients with recent stroke met the current APS criteria. If aCL, aβ2GPI, and aPS/PT of any isotype are included in the criteria, more than 30% of the stroke patients would be classified as APS.

J.O. Latino (Buenos Aires, Argentina) investigated the best time to assess the aPL profile for the prediction of the obstetric outcome in APS. Women diagnosed with obstetric APS prior to a new pregnancy (basal serological risk) were retested for aPL during the first trimester of pregnancy and treated with conventional therapy. Interestingly, risk stratification during the first trimester of pregnancy was found to be better in predicting pregnancy outcome compared with baseline measurements.

For the first time, during a meeting of the European Forum on Antiphospholipid Antibodies, a patient was invited to share his experience as APS patient and indicate the biggest unmet need. S. Otter (Patient organization NVLE, The Netherlands) illustrated from his own experience the importance of a faster and better diagnosis of APS. In his case, diagnosis took more than 6 years, leading to irreversible damage and a reduced quality of life. He also emphasized on the need for education and spreading of the knowledge about APS, not only for patient associations, but also for medical doctors.

## Scientific Session II: Clinical Features and Treatment of APS

R. Furie (NY, USA) presented a summary on the pathogenesis of APS, with a focus on aβ2GPI-β2GPI that bind to thrombi, enhancing platelet activation resulting in enhanced endothelium activation and fibrin formation. Thrombotic microangiopathy, movement disorder, and nephropathy may be associated with APS although these clinical manifestations are uncommon. There are still unmet needs in the treatment of APS, including risk stratification and safe therapy to treat asymptomatic patients at risk in primary thrombosis prevention. In secondary prevention, a safer alternative to warfarin is needed and patients refractory to anticoagulation are still challenging. Novel treatment strategies including immunologic approaches, novel anticoagulants, and novel anti-platelet agents were briefly discussed.

R. Cervera (Barcelona, Spain) looked back at the start of the European Forum in 1996 that already resulted in more than 100 collaborative research projects. Characteristics of two large multicenter studies, the Euro-Phospholipid project and CAPS Registry, were reviewed. Finally, the PRECISESADS project was introduced, aiming to find clinically useful biomarkers based on data gathered from 2500 people with various autoimmune diseases concerning the molecular cause of their disease.

H. Cohen (London, UK) presented an update on the RISAPS study, a randomized controlled phase 2/3 non-inferiority trial testing rivaroxaban (15 mg twice daily) versus warfarin (target INR 3.5) in 140 stroke patients with APS. Primary outcome (i.e., rate of change in brain white matter hyperdensity between baseline, and 24 months follow-up) as well as key secondary outcomes (i.e., neuroradiological markers, clinical events) will be compared.

S. Zuily (Nancy, France) proposed to create a European registry on DOACs use in APS, a project supported by the European Forum. Given the wide variety of APS manifestations and the existence of patients at high and low risk for clinical manifestations, it is unlikely that all APS patients can be efficiently treated with DOACs. The registry aims to identify the variables associated with thrombosis recurrence while on DOACs. Compared to a meta-analysis, a registry has the advantages to also include non-published data, to record data with a higher accuracy and result in less missing data.

A. Tincani (Brescia, Italy) proposed an important role for the European Forum on Antiphospholipid Antibodies in the European Reference Network (ERN). The ERN aims to tackle complex/rare diseases requiring specialized treatment and concentration of knowledge. More specifically, ReCONNET, the ERN on connective tissue and musculoskeletal diseases, includes APS. The European Forum may help to assemble the most reliable database including all the information necessary for patient’s management without being too time-consuming for clinicians. On the other hand, ReCONNET will provide a European Platform for the registry which will be disseminated to all health care providers.

A. Mekinian (Paris, France) proposed a prospective and retrospective multicenter open-labeled study to evaluate obstetrical outcome and treatments during pregnancy in seronegative primary APS patients. Included patient samples are required to meet thrombotic and/or obstetrical primary clinical seronegative APS (Sydney criteria) and the presence of at least one non-conventional aPL. The study is now open for other European centers to join.

C. Belizna (France) presented the first results on the retrospective part of the Hibiscus Study, i.e., the use of hydroxychloroquine (HCQ) to prevent thrombotic and obstetric relapses in primary APS. In the retrospective arm of the study, first results are encouraging. The prospective part of Hibiscus study, i.e., a double-blind randomized versus placebo multicenter trial for the use of HCQ to prevent relapses in primary APS, will start soon in France, but other centers are invited to join the project.

M. Radin (Turin, Italy) discussed the role of the laboratory tests in withdrawing anticoagulation when aPL turn negative. The withdrawal of anticoagulation when aPL turn negative remains difficult as in literature quite some recurrences have been reported after stopping anticoagulation. Questions that remain include if we should perform aPL testing in the follow-up of APS patients and how often aPL should be tested. Additionally, should these patients negative for criteria aPL be tested for non-criteria aPL or other tests such as thrombin generation for an improved risk classification? In preliminary data, patients that turned negative for aPL still showed a prothrombotic state in a thrombin generation assay.

S. Sciascia (Turin, Italy) continued on the therapeutic options when aPL turn negative. It remains to be determined whether in patients that turned aPL negative, vitamin K antagonist (VKA) therapy can be stopped while there is still a risk on recurrence. Would it be safer to switch VKA to DOACs given the lower risk of the patients? Given the poor level of evidence, a Delphi exercise was launched among the members of the European Forum aiming to achieve some level of consensus on this important topic.

In the abstract presentations of the second session, V. Dufrost (Nancy, France) performed a systematic review about the use of DOACs in APS, aiming to identify risk factors predisposing to thrombotic events. Among the 447 patients included in the analysis, high-risk (triple positive) APS patients were found to have a fourfold increased risk of thrombosis recurrence if treated with DOACs. Taken together, the results suggest that DOACs should be used with caution in APS patients and illustrate the need for randomized controlled studies.

M. Serrano (Madrid, Spain) demonstrated that immune complexes of β2GPI in IgA-positive patients identify patients with an elevated risk of early mortality after heart transplantation. Immune complexes of β2GPI-IgA proved to be an independent risk factor for mortality after heart transplantation. Given the small sample size of the study, further studies are needed to confirm the data.

N. Noirclerc (Nancy, France) investigated the clinical and laboratory associations and risk factors for progression of heart valve disease (HVD) in APS. About 40% of the included APS/systemic lupus erythematosus (SLE) patients suffered from HVD. SLE was found to be associated with HVD and renal disease was predictive for HVD progression. Young age and obstetrical morbidity on the other hand proved to be protective.

C. Ramírez (San Diego, USA) evaluated a novel multi-analyte assay for the detection of autoantibodies for the diagnosis of APS. The study showed excellent analytical and clinical performance of the novel multi-analyte assay measuring IgG/IgM/IgA aCL and aβ2GPI and/or aPS/PT as well as moderate-to-good correlation to the reference methods.

I. Cecchi (Turin, Italy) presented a validation study of GAPSS in women with SLE and pregnancy morbidity. Higher GAPSS values were found in women with SLE and aPL with previous pregnancy complications compared to those without pregnancy complications. The clinical utility of the GAPSS in pregnancy seems to be promising but validation in a prospective study is necessary.

D.E. Pleguezuelo (Madrid, Spain) investigated aPS/PT IgG and IgM antibodies in unexplained obstetric morbidity. Patients with recurrent reproductive failure displayed a high percentage (25%) of aPS/PT antibodies. Anti-PS/PT IgG and IgM antibodies were both strongly correlated with implantation failures and miscarriages compared to healthy pregnant women.

L. Stojanovich (Belgrade, Serbia) showed that the presence of immune complexes of IgG/IgM bound to β2GPI is associated with non-criteria manifestations in APS like thrombocytopenia and livedo reticularis, as well as with a higher complement consumption.

E. Fuzzi (Stockholm, Sweden) presented a study from the Karolinska University Hospital network in which characteristics of the aPL+/APS subgroup of consecutive SLE patients were assessed cross-sectionally. Patients with SLE and aPL antibodies were associated with complement consumption/activation, thrombocytopenia, hemolytic anemia, and higher SLE damage index scores than SLE patients without the persistent presence of aPL antibodies.

T. Lisitsyna (Moscow, Russia) evaluated mental disorders in patients with secondary APS (SAPS). Cognitive disorders were diagnosed more in patients with SAPS than in patients with SLE alone. Furthermore, patients with APS were more likely to have adverse childhood experiences, but with less stressful events before SLE, compared to patients without APS.

I. Cecchi (Turin, Italy) presented results on biological therapy in APS. In a retrospective study, female patients with primary APS (PAPS) and severe thrombocytopenia were treated with rituximab as a rescue therapy. Platelet levels normalized after treatment in five out of six patients after rituximab administration. In another retrospective study, disappearance of aPL was observed in SLE patients after starting therapy with belimumab. Despite the limited amount of data, targeting B cells seems to be a promising therapeutic option in the management of APS patients. Possibly, the current anti-thrombotic approach should be combined with an immunomodulatory approach.

## Scientific Session III: Pathophysiology of APS

P.L. Meroni (Milan, Italy) presented an update concerning the pathophysiology of APS and the associated impact on the clinical management. The localization of clotting instead of systemic coagulopathy is suggestive for a key pathogenic role for the endothelium rather than other cells. A second hit seems to be necessary to observe β2GPI colocalization with aPL in an affected vascular wall. The importance of complement activation in APS is supported by animal models, as C3, C5, and C6 knock-out mice were protected from thrombophilia induced by aPL and complement activation products are increased in APS patients. Results obtained from in vivo mouse models and from clinical studies support the existence of pathogenic (aDI) and protective (aDIV/aDV) aPL, illustrating the need for risk profiles. Finally, the difference between thrombotic and obstetric APS was discussed. Although most patients have both manifestations, pure variants exist. Considering the difference in pathology (thrombosis for vascular APS versus defective placentation for obstetric APS), it is unclear if the same (β2GPI-dependent) aPL induce both variants.

K. Lackner (Mainz, Germany) presented the role of aPL in cellular responses with respect to their epitope. Several human monoclonal aPL were isolated from APS patients, including lipid-reactive, cofactor-independent aPL that bind to CL but not to β2GPI. These aCL antibodies were found to accelerate thrombosis in vivo and activate monocytes. Monoclonal aCL and aβ2GPI aPL from the same APS patient had identical variable regions of the heavy chains and high similarity in the light chains. This suggests that aCL antibodies might convert to aβ2GPI antibodies by a limited number of somatic mutations. Interestingly, aPL with different binding specificity induce a pro-inflammatory and/or procoagulant effect by distinct mechanisms. The high risk of triple positivity may be related to the synergistic action of different pathogenic aPL.

S. Zuily (Nancy, France) evaluated whether APS is a thrombophilia or a vasculopathy. APS includes not only thrombosis and pregnancy morbidity, but also non-criteria manifestations are often seen in the same patients. These non-criteria manifestations are most likely due to vascular lesions which are irresponsive to anticoagulants. Therefore, APS is both a thrombophilia (durable hypercoagulable state) and a vasculopathy (disease affecting blood vessels).

V. Pengo (Padua, Italy) presented the role of platelets and thrombocytopenia in APS. Some studies indicate that β2GPI-IgG complexes induce platelet activation. Platelet count is often in the normal range in high-risk triple positive patients with APS. However, a drop in platelet count in those patients may indicate platelet consumption/deposition in the microcirculation with consequent organ failure as a result (CAPS).

E. Svenungsson (Stockholm, Sweden) presented the role of microparticles in APS. Microparticles (MP) are formed through a blebbing process, carrying markers from its parental cell and procoagulant and pro-inflammatory molecules. Patients with SLE were found to have an increased number of MP compared to healthy controls. TF-expressing monocyte MP were found to be increased in APS patients. Both IgG and β2GPI were upregulated on MP from SLE patients. It was hypothesized that aβ2GPI bind to β2GPI on MP of APS/SLE patient and shield the MP, preventing rapid clearance and activating complement/inflammation.

H.C. Hemker (Maastricht, the Netherlands) evaluated the importance of phospholipids (PL) in LAC testing. Clotting and thus LAC depend on the nature of PL surfaces. To improve the reproducibility of LAC tests, well-defined PL should be used. The use of a patient’s own PL such as platelets may give more insight in what happens in that specific patient. Finally, different PL can be used to study inhibitory actions of aPL and understand their mechanism.

In the abstract presentations of the third session, C. Grossi (Milan, Italy) gave new insights into APS as antibodies to DV of β2GPI fail to induce thrombi in rats. However, aDI serum IgG induced blood clotting in rats injected with lipopolysaccharide. Interestingly, aDV IgG antibodies do not recognize CL-bound β2GPI, but interact with β2GPI in the fluid phase, possibly providing an explanation why aDV IgG fail to induce thrombosis in vivo. The ratio aDI/aDV may be a useful biomarker for risk stratification in APS patients.

P.A. Lonati (Milan, Italy) evaluated the importance of complement activation in APS by measuring platelet-bound C4d in ex vivo and in vitro studies. The ex vivo association of aPL with platelet-bound C4d suggests the involvement of the classic complement pathway in the pathogenesis of APS. In vitro data support this hypothesis. In the presence of a second hit, β2GPI was able to bind to platelets, followed by aβ2GPI. The resulting immune complex then activates the complement, leading to C4d deposition.

N. Sippl (Stockholm, Sweden) characterized β2GPI specific B cells from APS patients. The number of β2GPI-specific B cells, especially plasmablasts, was increased in APS patients. Of note, the VH gene usage showed a bias towards different V-regions depending on the isotype. Recombinant antibodies are currently being produced to further investigate the binding and pathogenic properties.

T. Moulinet (Nancy, France) evaluated the predictive value of thrombocytopenia in APS. In a prospective study including aPL-positive patients with or without SLE, thrombocytopenia proved to be a strong predictor of thrombosis and obstetrical events. Based on these data, thrombocytopenia may be added to risk scores to improve risk stratification.

## Symposium on Novel Biomarkers for APS: Opportunities for Improving Diagnosis and Beyond

C.B. Chighizola (Milan, Italy) presented that aDI antibodies identify late pregnancy morbidity in APS. A strong association of aDI antibodies with late pregnancy morbidity was found but not with early pregnancy morbidity. Interestingly, reactivity to DIV/V was not associated with thrombosis/pregnancy morbidity. In contrast to early pregnancy morbidity, late pregnancy morbidity responds poorly to treatment with low-dose aspirin and low molecular weight heparin.

S. Sciascia (Turin, Italy) presented the reliability of LAC and aPS/PT results and their impact on the clinical management of APS patients. Samples of thrombotic patients were sent to four centers and tested for LAC and aPS/PT. Whereas aPS/PT showed a good agreement, a high level of disagreement was observed for LAC. The introduction of aPS/PT in the diagnostic process of APS within the current criteria may be valuable, especially when LAC is not available or unreliable (mainly because of anticoagulation therapy).

A. Hoxha (Padua, Italy) evaluated the diagnostic value of novel biomarkers for predicting clinical complications in aPL asymptomatic patients. Anti-β2GPI IgA were found to be an independent risk factor for the development of thrombotic events in asymptomatic subjects. In addition, aDI and aPS/PT IgG antibodies were independent risk factors for the development of thrombotic events in aPL carriers. Detection of IgA-aβ2GPI, aDI, and aPS/PT antibodies may therefore be used for risk assessment along with conventional tests.

**Fig. 1:**
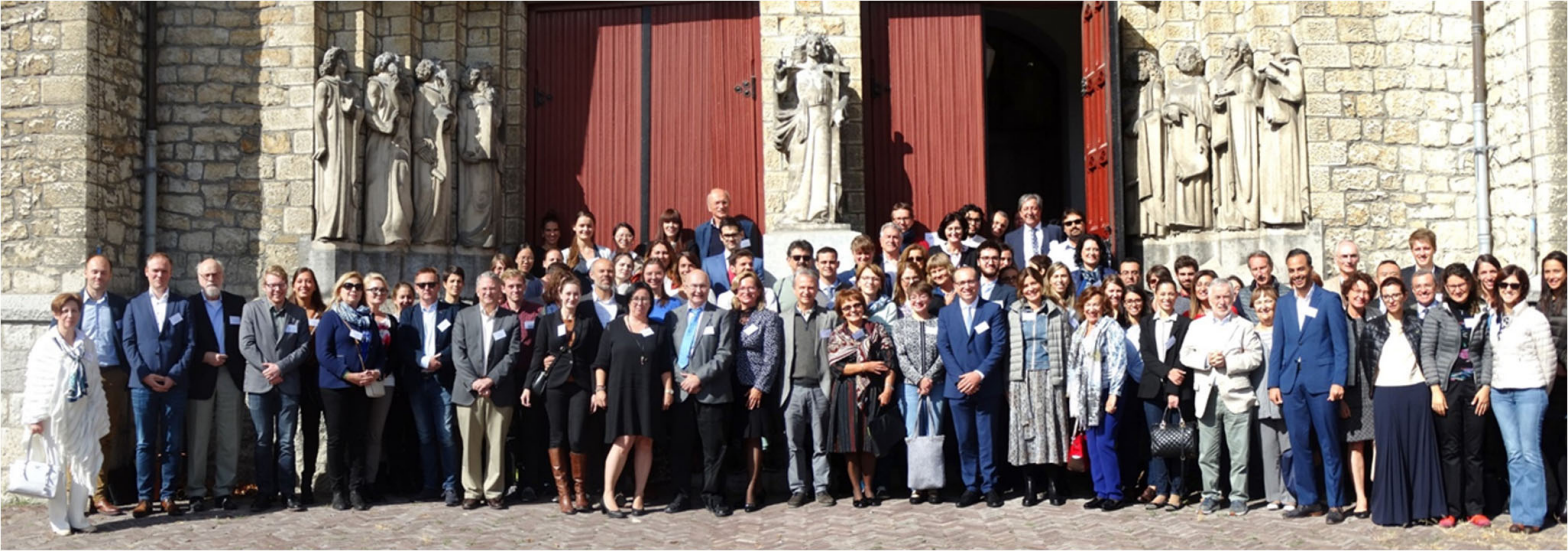
ᅟPhoto taken during the 11th Meeting of the European Forum on Antiphospholipid Antibodies

### Key Messages

- The 11th meeting of the European Forum on Antiphospholipid Antibodies was held in Maastricht, The Netherlands, in September 2018. Some of the presentations are available for educational purposes at https://apsmaastricht.com
- The biggest unmet need from a patient’s perspective is a faster and better diagnosis of the antiphospholipid syndrome (APS).
- Important ongoing European projects and new findings were presented: the possible added value of criteria and non-criteria antiphospholipid antibodies (aPL); the use of DOAC-stop to avoid false positive results in lupus anticoagulant testing; the need to better standardize laboratory assays; DOAC use in APS patients with updates on the clinical trials and proposal for registry; the possible withdrawal of anticoagulation when aPL turn negative; possibilities for new therapeutic options such as combined anticoagulant and immunomodulatory therapy; the existence of pathogenic versus non-pathogenic antibodies with opportunities for risk profiles.
- An important participation of the European Forum on Antiphospholipid Antibodies in the European Reference Network ReCONNET was proposed.
- The next meeting of the European Forum on Antiphospholipid Antibodies will take place in Belgrade, Serbia, in the spring of 2020.

